# Optimization and validation of RT-LAMP assay for diagnosis of SARS-CoV2 including the globally dominant Delta variant

**DOI:** 10.1186/s12985-021-01642-9

**Published:** 2021-08-30

**Authors:** Vijay Lakshmi Jamwal, Natish Kumar, Rahul Bhat, Piyush Singh Jamwal, Kaurab Singh, Sandeep Dogra, Abhishek Kulkarni, Bhaskar Bhadra, Manish R. Shukla, Saurabh Saran, Santanu Dasgupta, Ram A. Vishwakarma, Sumit G. Gandhi

**Affiliations:** 1 CSIR-Indian Institute of Integrative Medicine, Canal Road, Jammu, 180001 India; 2 Academy of Scientific and Innovative Research (AcSIR), Ghaziabad, 201002 India; 3 Higher Education Department, Union Territory of Jammu and Kashmir, Jammu, India; 4 Department of Microbiology, Government Medical College, Jammu, 180001 India; 5 A2O - Biology, Reliance Technology Group, Reliance Industries Limited, RCP, Navi Mumbai, 400701 India

**Keywords:** B.1.1.7 (Alpha), B.1.351 (Beta), P.1 (Gamma), B.1.617.2 (Delta), B.1.427, B.1.429 (Epsilon), P.2 (Zeta), B.1.525 (Eta), P.3 (Theta), B.1.526 (Iota), B.1.617.1 (Kappa), COVID-19, Loop-mediated isothermal amplification, Molecular diagnosis, RT-LAMP, SARS-CoV-2

## Abstract

**Background:**

Severe Acute Respiratory Syndrome Coronavirus 2 (SARS-CoV-2), the causative agent of COVID-19 pandemic, has infected more than 179 million people worldwide. Testing of infected individuals is crucial for identification and isolation, thereby preventing further spread of the disease. Presently, Taqman™ Reverse Transcription Real Time PCR is considered gold standard, and is the most common technique used for molecular testing of COVID-19, though it requires sophisticated equipments, expertise and is also relatively expensive.

**Objective:**

Development and optimization of an alternate molecular testing method for the diagnosis of COVID-19, through a two step Reverse Transcription Loop-mediated isothermal AMPlification (RT-LAMP).

**Results:**

Primers for LAMP were carefully designed for discrimination from other closely related human pathogenic coronaviruses. Care was also taken that primer binding sites are present in conserved regions of SARS-CoV2. Our analysis shows that the primer binding sites are well conserved in all the variants of concern (VOC) and variants of interest (VOI), notified by World Health Organization (WHO). These lineages include B.1.1.7, B.1.351, P.1, B.1.617.2, B.1.427/B.1.429, P.2, B.1.525, P.3, B.1.526 and B.1.617.1. Various DNA polymerases with strand displacement activity were evaluated and conditions were optimized for LAMP amplification and visualization. Different LAMP primer sets were also evaluated using synthetic templates as well as patient samples.

**Conclusion:**

In a double blind study, the RT-LAMP assay was validated on more than 150 patient samples at two different sites. The RT-LAMP assay appeared to be 89.2% accurate when compared to the Taqman™ rt-RT-PCR assay.

**Supplementary Information:**

The online version contains supplementary material available at 10.1186/s12985-021-01642-9.

## Introduction

Severe Acute Respiratory Syndrome (SARS) is a respiratory disease caused by a coronavirus that appears to have jumped from horseshoe bats to humans [1, 2]. SARS outbreak, caused by the SARS-CoV, occurred during 2002–2004 [3]. A similar respiratory disease caused by another coronavirus, which originated in the Middle East, resulted in an outbreak during the 2012–2013 and was named Middle East Respiratory Syndrome (MERS). MERS is caused by a MERS-CoV, which is believed to have jumped from bats to camels and then infect humans [4, 5]. Recently, towards the end of 2019 another coronavirus, named SARS-CoV-2 resulted in an outbreak of Coronavirus Disease 2019 (COVID-19). Since the outbreak, COVID-19 pandemic has infected more than 179 million people worldwide resulting in death of about 3.9 million people. Few countries such as Israel and others from the Western Pacific Region have been able control the outbreak significantly (https://covid19.who.int/). Vaccination, social distancing, general hygiene, contact tracing and most importantly, diagnostic testing for COVID-19 in high numbers, are considered to be important factors in controlling spread of the disease [6, 7]. Transmissibility of SARS-CoV-2 was found to be about 55% with approximately 30% of individuals remaining asymptomatic [8]. However, new variants, such as the Delta appears to exhibit prominently higher transmissibility attributed to mutations in the receptor binding domain of spike protein [9]. This necessitates the testing of as many individuals as possible to diagnose those infected with SARS-CoV-2 and more importantly to identify the asymptomatic carriers who may unknowingly infect a large population. SARS-CoV-2 is a single-stranded positive-sense RNA virus with approximately 29.9 kb genome size [10]. The capsid outside the genome is formed of nucleocapsid protein (N) which is further enclosed by an envelope. Three types of structural proteins are associated with the envelope: spike protein (S), membrane protein (M) and envelope protein (E). Other than these four structural proteins (N, S, M and E) SARS-CoV-2 genome also encodes sixteen non-structural proteins (NSP1-16). RNA dependent RNA polymerase (RdRp) also known as NSP 12, plays an important role in multiplication of coronavirus [11]. In late 2020, a fast-spreading B.1.1.7 strain was reported which has mutation in spike protein and 56% more transmissible than the earlier reported strain of SARS-CoV2 [12]. Another fast-spreading B.1.351 variant of SARS-CoV-2 was reported from South Africa. P.1, P.2 and P3 variants were reported from Brazil. B.1.427/B.1.429 variant was first identified in California. The first case of B.1.525 variant was reported in UK. B.1.526 variant was originated in New York. Recently in India, SARS-CoV-2 variants were reported that belongs to the B.1.617 lineage: B.1.617.1 and B.1.617.2 (https://www.who.int/en/activities/tracking-SARS-CoV-2-variants/). WHO keeps a close watch on the transmissibility and spreading of new variants of SARS-CoV-2. During late 2020, WHO categorized the emerging variants of SARS-CoV-2 as Variants of interest (VOIs) and Variants of concern (VOCs). The variants with increased transmissibility that can change epidemiology of COVID-19 and/or increased virulence that can reduce the effectiveness of vaccines, diagnosis, therapeutics, etc. are termed as Variants of interest (VOIs) whereas the variants that increase the severity of the disease and results in community transmission/multiple COVID-19 cases/clusters, or has been detected in multiple countries are termed Variants of concern (VOCs). B.1.1.7 (Alpha), B.1.351 (Beta), P.1 (Gamma), B.1.617.2 (Delta) are presently grouped in VOCs, while B.1.427/B.1.429 (Epsilon), P.2 (Zeta), B.1.525 (Eta), P.3 (Theta), B.1.526 (Iota), B.1.617.1 (Kappa) are mentioned as VOIs (https://www.who.int/en/activities/tracking-SARS-CoV-2-variants/).

Serological or nucleic acid detection based assays may be used for the diagnosis of COVID-19. Serological assays mostly depend on the detection of viral particles in nasal or throat swab samples by using antibodies against the spike protein. Such assays provide a quick diagnosis with minimalistic infrastructure and expertise [13, 14]. However, such rapid antigen tests suffer from a high false negative rate [15, 16]. Hence nucleic acid based assays remain the popular choice [17].

Reverse transcription real-time PCR (rt-RT-PCR) employing the Taqman™ chemistry for detection of viral RNA is considered the gold standard for diagnostic testing of individuals for COVID-19 [17]. A validated rt-RT-PCR assay for COVID-19 diagnosis was designed at Charité Institute of Virology in Berlin, Germany [18]. Similar assays have been described in many other countries and are commercially available through several companies [19]. This involves Taqman™ probe based assay for the E gene, followed by a Taqman™ assay for RdRp gene for confirmation. Hong Kong University developed a Taqman™ probe based assay for the N gene followed by a confirmatory assay for Orf1b. United States Centres for Disease Control (US-CDC) has designed Taqman™ rt-RT-PCR using 3 primer–probe assays (N1, N2 and RP), which are assayed separately but simultaneously (https://www.who.int/docs/default-source/coronaviruse/whoinhouseassays.pdf).

Worldwide, the high demand for Taqman™ assays and other reagents required for the test has resulted in higher prices and shortages, especially for the developing countries with a large population [20]. Dye based rt-RT-PCR assay using SYBR Green I have been proposed by several research groups for the diagnosis of COVID-19 [21–24]. Next Generation Sequencing (NGS) based assay for testing hundreds to thousands of patient samples in parallel have also been proposed and are under development. Recently, one such NGS based assay for parallel testing of COVID-19 has been cleared for marketing by the United States Food and Drug Administration (US-FDA) under Emergency Use Authorization (EUA) (https://www.fda.gov/media/146933/download). Reverse Transcription Loop-Mediated Isothermal Amplification (RT-LAMP) is also a viable alternative for diagnostic detection of COVID-19 and has been proposed and developed by several researchers. RT-LAMP assays rely on conversion of viral RNA to cDNA, followed by target DNA amplification using a set of four to six primers, at a uniform temperature, without the requirement of temperature cycling. A large amount of target DNA is rapidly amplified in a LAMP reaction and can be detected using DNA binding dyes such as SYBR Green I or through changes in pH, which can be detected visually or colorimetrically by using pH sensitive dyes [25, 26]. RT-LAMP assays have been described for several human pathogenic viruses, including the Influenza viruses, Zika virus, Chikungunya virus, West Nile virus, etc. [27–30].

## Materials and methods

### Collection of samples and isolation of viral RNA

Naso-pharyngeal swabs were collected and stored in Viral Transport Medium (VTM) as per the standard procedures [31]. Total RNA was isolated from VTM using QIAamp Viral RNA Mini Kit (QIAGEN, Germany), following the manufacturer’s instructions. RNA was quantified using the NanoDrop™ spectrophotometer (NanoDrop 2000c, Thermo Fisher Scientific, USA). Control human RNA for generating synthetic template of β-Actin was isolated from PANC-1 cell lines maintained in DMEM supplemented with 10% FBS and 1% penicillin–streptomycin, at 37 °C under 5% CO_2_, as described earlier [32]. The quality and quantity of control RNA was assessed by running on 2% agarose gel and measuring optical density (OD) at 260 nm using NanoDrop™ spectrophotometer, respectively.

### Taqman™ rt-RT-PCR

5 µL of viral RNA was added to a 20 µL reaction mixture containing 12.5 µL of 2X reaction mix, 1.5 µL Primer–Probe mix, 0.5 µL of Superscript™ III RT/Platinum^TM^*Taq* Mix (Thermo Fisher Scientific, USA) and 5.5 µL nuclease free water. Reactions were carried out in 7500 Real-Time PCR (Applied Biosystems, Thermo Fisher Scientific, USA) or Rotor-Gene Q 2Plex (QIAGEN, Germany) with the following thermal cycling conditions: incubation at 50 °C for 30 min, 95 °C for 3 min followed by 45 two-step cycles of 95 °C for 15 s and 58 °C for 30 s, with data collection in green channel at the annealing-extension step. A positive viral template control (VTC) and a negative no template control (NTC) assay were carried out in each run. For VTC, 1.5 µL of control templates (positive control kits obtained from Integrated DNA Technologies, USA) were used, while for NTC, nuclease free water was used instead of template.

### Reverse transcription loop-mediated isothermal amplification

cDNA was synthesized in a 10 µL RT reaction, by using ImProm-II™ Reverse Transcription System (Promega, USA) with random hexamers and using 10–40 ng of viral RNA and control human RNA (PANC-1) as template, following manufacturer’s instructions. cDNA was used immediately for LAMP reaction or stored at − 40 °C until further use. cDNA was also used for generating synthetic templates for viral genes as well as for control human β-Actin gene. 10 µL LAMP reaction contained 1 µL cDNA or 1–2 ng of synthetic template, 1µL of 10X LAMP primer mix, 1 µL of 2 mM dNTP, 1 µL of 10X buffer, 0.3–1 µL (2.4–10 U) of LAMP DNA Polymerase and nuclease free water to make up the reaction volume. Additionally, MgSO_4_ or MgCl_2_, were also used in reactions with some LAMP Polymerases and the optimized conditions are discussed in results section. Reactions were always set in a cold block. Reactions were incubated at uniform temperature of 50 °C to 60 °C, depending on the DNA polymerase enzyme used for LAMP reaction. Quantities of the DNA oligos used in 10X LAMP primer mix were optimized for each of the LAMP primer set and have been described in the results section.

### Visualization, densitometry and statistical analysis

At the end of LAMP reaction, an appropriate quantity of SYBR Green 1 dye (0.2–1 µL of 1:100 dilution in DMSO) was added and the tubes were visually examined under UV light and photographed using a 8 MP auto-focus digital camera with ISO 1600-3200 and exposure 1/15 to 1/30 s. Integrated density of the fluorescence in the reaction tubes was measured using the measurement tool in Photoshop CS3 extended (Adobe, USA) and for calculation integrated density of NTC was subtracted from the integrated density of samples. For the measurement of densitometric ratios, the region of interest (ROI) was marked using the ‘lasso tool’. The ROI was marked in the area of tubes where reaction mixture was present and green fluorescence appeared. The size and shape of the ROI was kept constant for all tubes, including the NTC. Three technical replicates were used for analysis. Average and standard deviation calculations as well as plotting of graphs were done in MS Office Excel 2007 (Microsoft Corporation, USA). The reactions of RT-LAMP assays were also checked on 2% agarose gel for the presence of ladder pattern.

### Computational methods

Sequences of SARS-CoV2 and other betacoronaviruses were downloaded from the NCBI GenBank database (www.ncbi.nlm.nih.gov). Accession numbers of sequences are available in Sheet 1 of Additional file 1. Alignments were done using CLC Genomics Workbench 10 (Qiagen, Germany). LAMP primers were designed using PrimerExplorer V5 (https://primerexplorer.jp/e/).

## Results

### LAMP primers

**Fig. 1:**
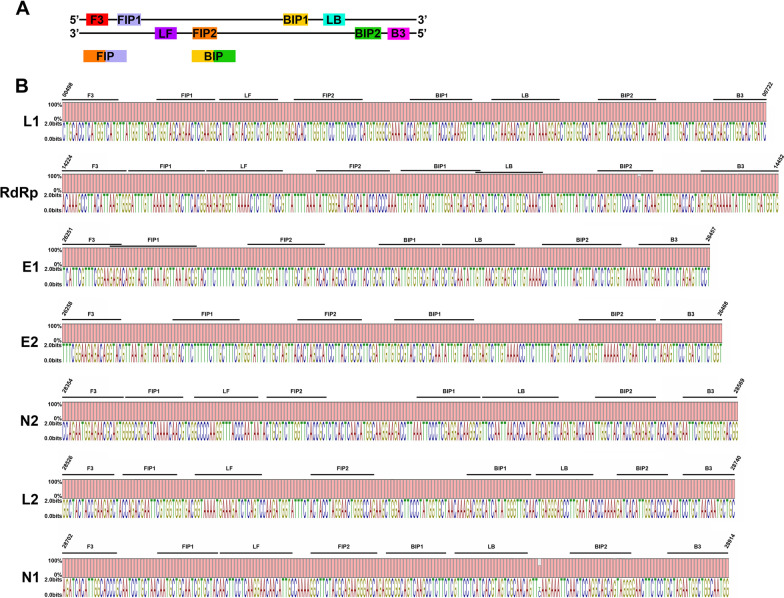
Conservation of LAMP primer binding sites in SARS-CoV2 genome. **a** Schematic depiction of design of LAMP primers. **b** Conservation graphs and Sequence Logo of LAMP primer binding sites

Four to six lamp primers were designed (Fig. 1a) for each of the seven loci (Fig. 1b, Table 1) from the viral genome (SARS-CoV2 reference genome sequence NC_045512.2) and two loci in the human β-actin gene (NM_001101.5). In order to ensure that the regions used for designing the LAMP primers are specific to SARS-CoV2, these sequence regions were analyzed by BLASTN (https://blast.ncbi.nlm.nih.gov). For this purpose all the reference sequences (NCBI GenBank RefSeq) that were available for betacoronaviruses (Sheet 1 of Additional file 1), including that of SARS-CoV2, were downloaded and a local BLAST database was created. Selected sequence regions of the SARS-CoV2, from where the LAMP primers were designed, were compared with the sequences of other betacoronaviruses (Additional file 1). As expected the second top hit for all primer binding regions was the SARS-CoV (Additional file 1). However, the percentage identity was never more than 93%. Further, alignments of the selected seven loci of SARS-CoV2, with the closest BLAST matches in the betacoronavirus genomes, showed significant differences in the primer binding sites. These alignments also suggest that L1, RdRp and N1 primer sets can discriminate between SARS-CoV and SARS-CoV2, due to significant sequence differences in binding sites of at least one or both of the outer primers (F3 and B3), that are critical for LAMP amplification. On the other hand, conservation of primer binding sites among the variants of SARS-CoV2 is vital for its detection. 284 complete genomes of SARS-CoV2 from India and 5642 genomes worldwide were surveyed. Sequences for the selected seven loci (Table 1, Fig. 1b), where LAMP primer were designed, were extracted from the complete genomes and multiple sequence alignments were carried out for each region. In L1 region, although variants were observed in 54 out of 225 nucleotides in the 5642 completed SARS-CoV2 genomes; minimum conservation at any nucleotide position was 99.5% (Table 2). Results from similar analysis with other selected loci from SARS-CoV2 are summarized in Table 2. Nearly 1000 SARS-CoV2 genomes were sequenced from India, at the time of the study and the sequence variants and their percentages are available (http://data.ccmb.res.in/gear19/variants). These variants were mapped onto the selected sequence loci from where LAMP primers were designed (Additional file 2). As may be observed from the conservation graphs (Fig. 1b), multiple sequence alignments and mapping of identified sequence variants (Additional file 2), the primer binding sites were conserved in the selected loci. The minimum percentage of conservation in variant nucleotides in the L1 and L2 primer binding sites was 99% (Table 2).

**Table 1:** LAMP primer sequences

Gene	Protein	Genomic coordinates		Length of region	Name of primer set	Name of primer	Sequence
		Start	End				
ORF1AB	NSP1	498	722	225	L1	L1-F3	CTGCACCTCATGGTCATG
						L1-B3	GATCAGTGCCAAGCTCG
						L1-FI	GAGGGACAAGGACACCAAGTGTGGTAGCAGAACTCGAAG
						L1-C-BI	CAGTGGCTTACCGCAAGGTTTTAGATCGGCGCCGTAAC
						L1-LF	CACTACGACCGTACTGAAT
						L1-LB	CGTAAGAACGGTAATAAAGGAG
ORF1AB	RdRP	14,224	14,452	229	RdRp	rdrp1F3	ACAAAGCCTTACATTAAGTGG
						rdrp1B3	CACCATCAACAAATATTTTTCTCAC
						rdrp1FIP	TGGGTGGTATGTCTGATCCCAATAGATTTGTTAAAATATGACTTCACGG
						rdrp1BIP	TGTGTTAACTGTTTGGATGACAGATGTAGGTGGGAACACTGT
						rdrp1LF	CGGTCAAAGAGTTTTAACCTCTCTT
						rdrp1LB	TGCATTCTGCATTGTGCAAACT
E	E	26,251	26,457	207	E1	e1F3	TCATTCGTTTCGGAAGAGA
						e1B3	AGGAACTCTAGAAGAATTCAGAT
						e1FIP	TGTAACTAGCAAGAATACCACGAAACAGGTACGTTAATAGTTAATAGCG
						e1BIP	GCTTCGATTGTGTGCGTACTCGAGAGTAAACGTAAAAAGAAGG
						e1LB	GCTGCAATATTGTTAACGTGAGTC
E	E	26,258	26,468	211	E2	e2F3	TTTCGGAAGAGACAGGTAC
						e2B3	ACCAGAAGATCAGGAACTCT
						e2FIP	GCGCAGTAAGGATGGCTAGTGGTACTTCTTTTTCTTGCTTTCG
						e2BIP	TGCGTACTGCTGCAATATTGTTAACGAAGAATTCAGATTTTTAACACGAG
N	N	28,354	28,569	216	N2	n2F3	CCAGAATGGAGAACGCAGTG
						n2B3	CCGTCACCACCACGAATT
						n2FIP	AGCGGTGAACCAAGACGCAGGGCGCGATCAAAACAACG
						n2BIP	AATTCCCTCGAGGACAAGGCGAGCTCTTCGGTAGTAGCCAA
						n2LF	TTATTGGGTAAACCTTGGGGC
						n2LB	TTCCAATTAACACCAATAGCAGTCC
N	N	28,526	28,740	215	L2	L2-F3	GGCTACTACCGAAGAGC
						L2-B3	GCAGCATTGTTAGCAGG
						L2-FI	CTGGCCCAGTTCCTAGGTAGTCCAGACGAATTCGTGGTG
						L2-BI	GACGGCATCATATGGGTTGCACGGGTGCCAATGTGATC
						L2-LF	GACTGAGATCTTTCATTTTACC
						L2-LB	CTGAGGGAGCCTTGAATAC
N	N	28,702	28,914	213	N1	n1F3	AGATCACATTGGCACCCG
						n1B3	CCATTGCCAGCCATTCTAGC
						n1FIP	TGCTCCCTTCTGCGTAGAAGCCAATGCTGCAATCGTGCTAC
						n1BIP	GGCGGCAGTCAAGCCTCTTCCCTACTGCTGCCTGGAGTT
						n1LF	GCAATGTTGTTCCTTGAGGAAGTT
						n1LB	GTTCCTCATCACGTAGTCGCAACA
Human β-actin	Human β-actin	585	787	203	ACTB1	hACTBF3	GCGCGGCTACAGCTTCA
						hACTBB3	GGAAGAGTGCCTCAGGGC
						hACTBFIP	AAGTCCAGGGCGACGTAGCACCGGCCGAGCGGGAAAT
						hACTBBIP	GAGATGGCCACGGCTGCTTCCATTGCCAATGGTGATGACCT
						hACTBLF	TTCTCCTTAATGTCACGCACG
						hACTBLB	CCCTGGAGAAGAGCTACGAG
Human β-actin	Human β-actin	197	390	194	ACTB2	hACTB2F3	CCCTGAAGTACCCCATCGA
						hACTB2B3	TGGGGTGTTGAAGGTCTCAA
						hACTB2FIP	AGCCACACGCAGCTCATTGTAGCACGGCATCGTCACCAAC
						hACTB2BIP	AGCACCCCGTGCTGCTGAGTCATCTTCTCGCGGTTGG
						hACTB2LF	AGATTTTCTCCATGTCGTCCCA

**Table 2:** Conservation of LAMP primer binding sites in genomes of SARS-CoV2

Locus (primer set)	Length	Completed genomes worldwide (5642 sequences)		Completed Genomes from India (284 sequences)	
		Number of variant nucleotides	Minimum conservation in variant nucleotides (%)	Number of variant nucleotides	Minimum conservation in variant nucleotides (%)
L1	225	54	99.5	5	99.6
RdRp	229	24	64.2	4	87.4
E1	207	8	99.8	1	99.7
E2	211	7	99.9	1	99.7
N2	216	6	99.3	4	99.6
L2	215	14	99.1	8	99.3
N1	213	41	91	11	64

### Evaluation of DNA polymerases for LAMP

**Fig. 2:**
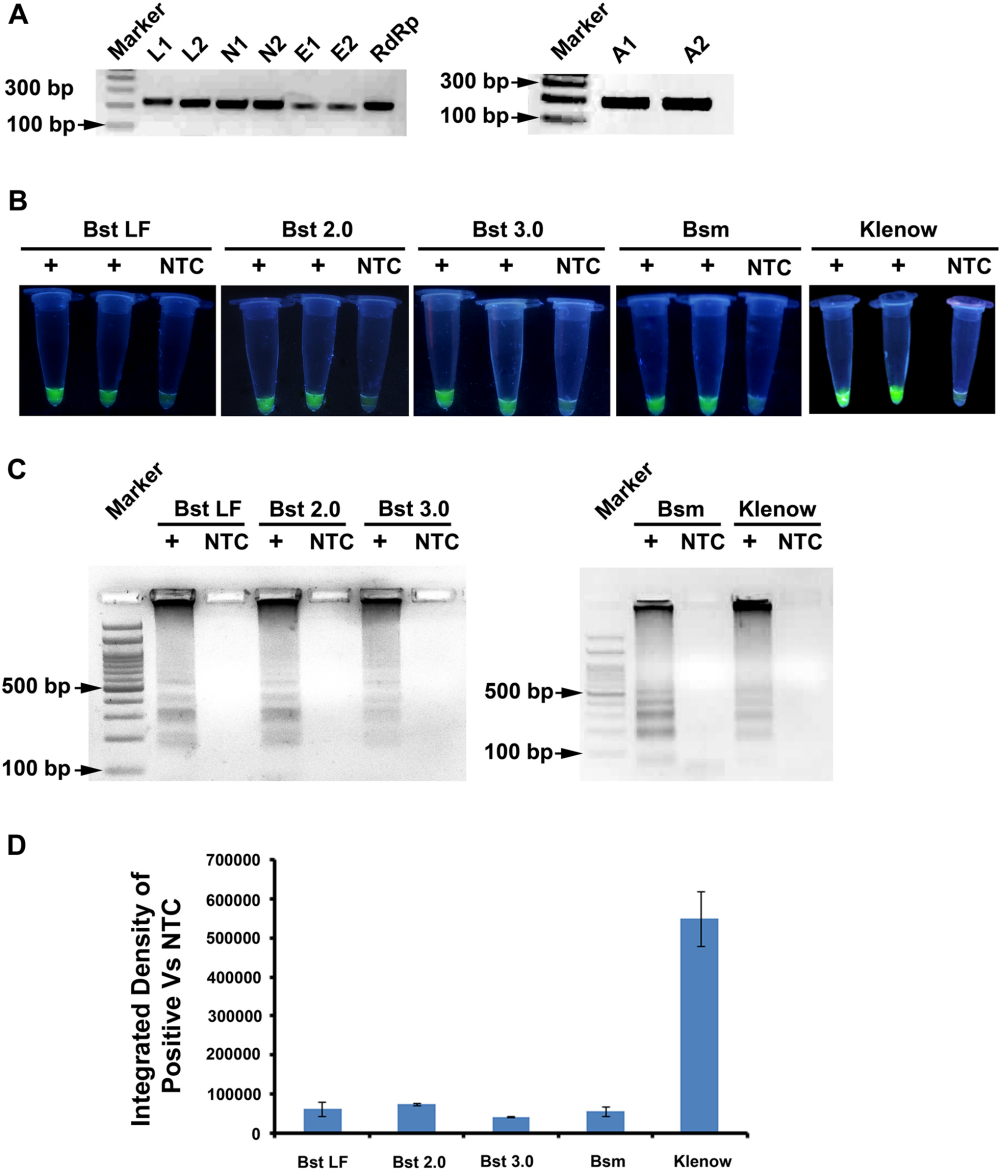
Evaluation of DNA polymerases for LAMP. **a** Generation of synthetic templates for LAMP from cDNA synthesized using viral RNA and human RNA. **b** Fluorescence imaging of LAMP assays with different DNA polymerases. **c** Agarose Gel Electrophoresis of LAMP amplicons showing ladder pattern for positive reaction. **d** Densitometry of fluorescence values from LAMP assays measured using **b**

In order to test LAMP primers, synthetic positive templates were prepared (Fig. 2a). Viral RNA or control human RNA were reverse transcribed with random hexamers, as described earlier [33]. Outer primers (F3 and B3; Fig. 1a) from each of the LAMP primer set (Table 1) were used for amplifying the target regions using cDNA as template. The amplified products were purified through gel elution and either used directly or cloned in PCR cloning vector and used as positive control template in LAMP reactions. Synthetic templates from viral loci were prepared from viral RNA while synthetic templates for β-actin were prepared from control human RNA. Five different DNA polymerases (Bst LF, Bst 2.0, Bst 3.0, Bsm and Klenow) were tested for LAMP and reaction conditions were optimized (Additional file 3). A positive LAMP reaction, identified by green fluorescence under the UV light, resulted when a synthetic positive control template was used, while the no-template-controls (NTC) lacked the fluorescence (Fig. 2b). This was further confirmed by electrophoresing the amplicons on an agarose gel, where only the positive LAMP reaction resulted in the classical ladder pattern (Fig. 2c). The fluorescence of the reaction tubes in Fig. 2b were analysed through densitometry. Densitometric analysis of fluorescence values showed that Klenow gave the best result (Fig. 2d).

### Evaluation of LAMP primer sets

**Fig. 3:**
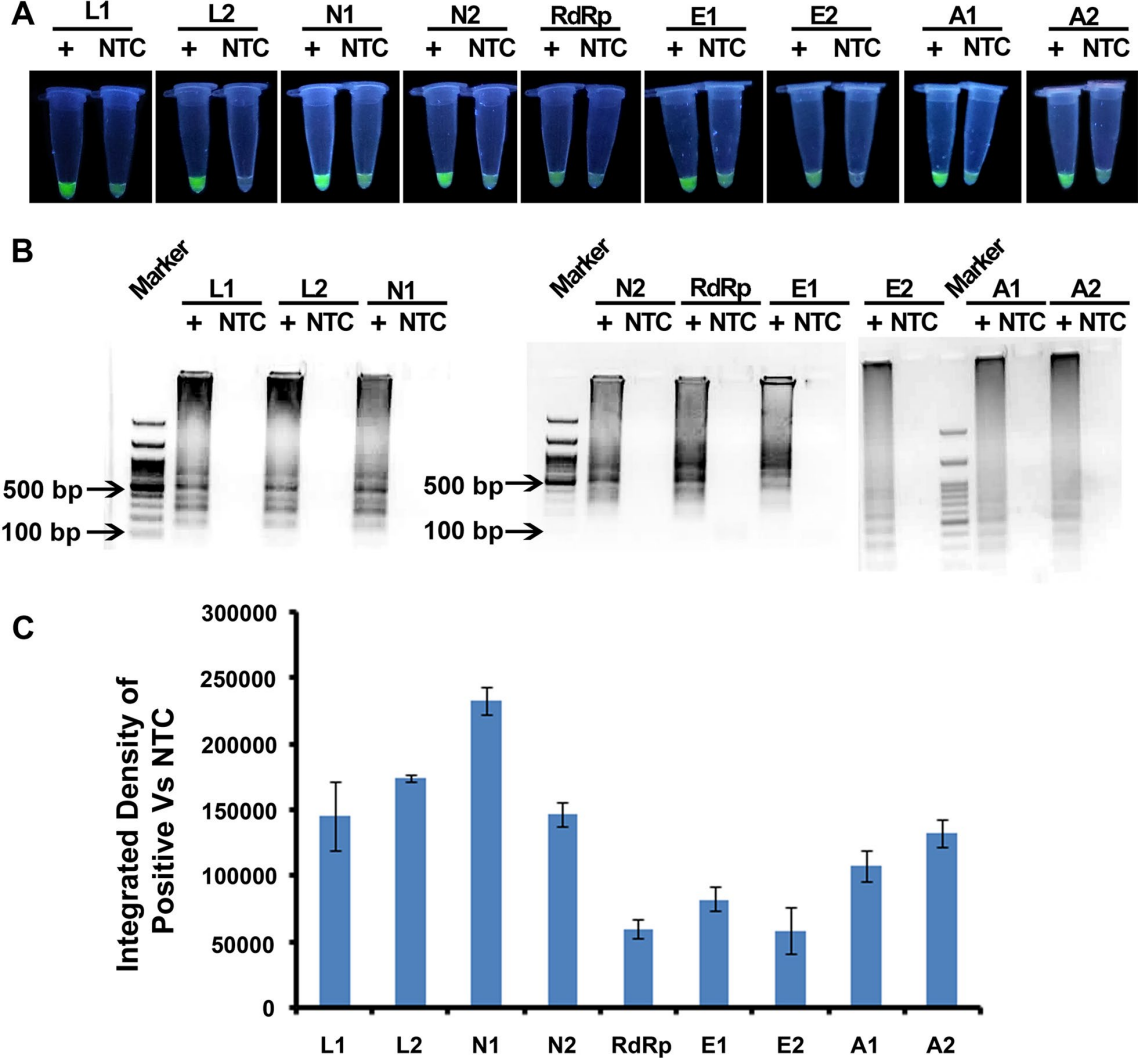
Evaluation of LAMP Primers. **a** Fluorescence imaging of LAMP assays of seven primer sets from viral genome and two β-actin primer sets. **b** Agarose Gel Electrophoresis of LAMP amplicons showing ladder pattern for positive reaction. **c** Densitometry of fluorescence values from LAMP assays measured using **a**

**Fig. 4:**
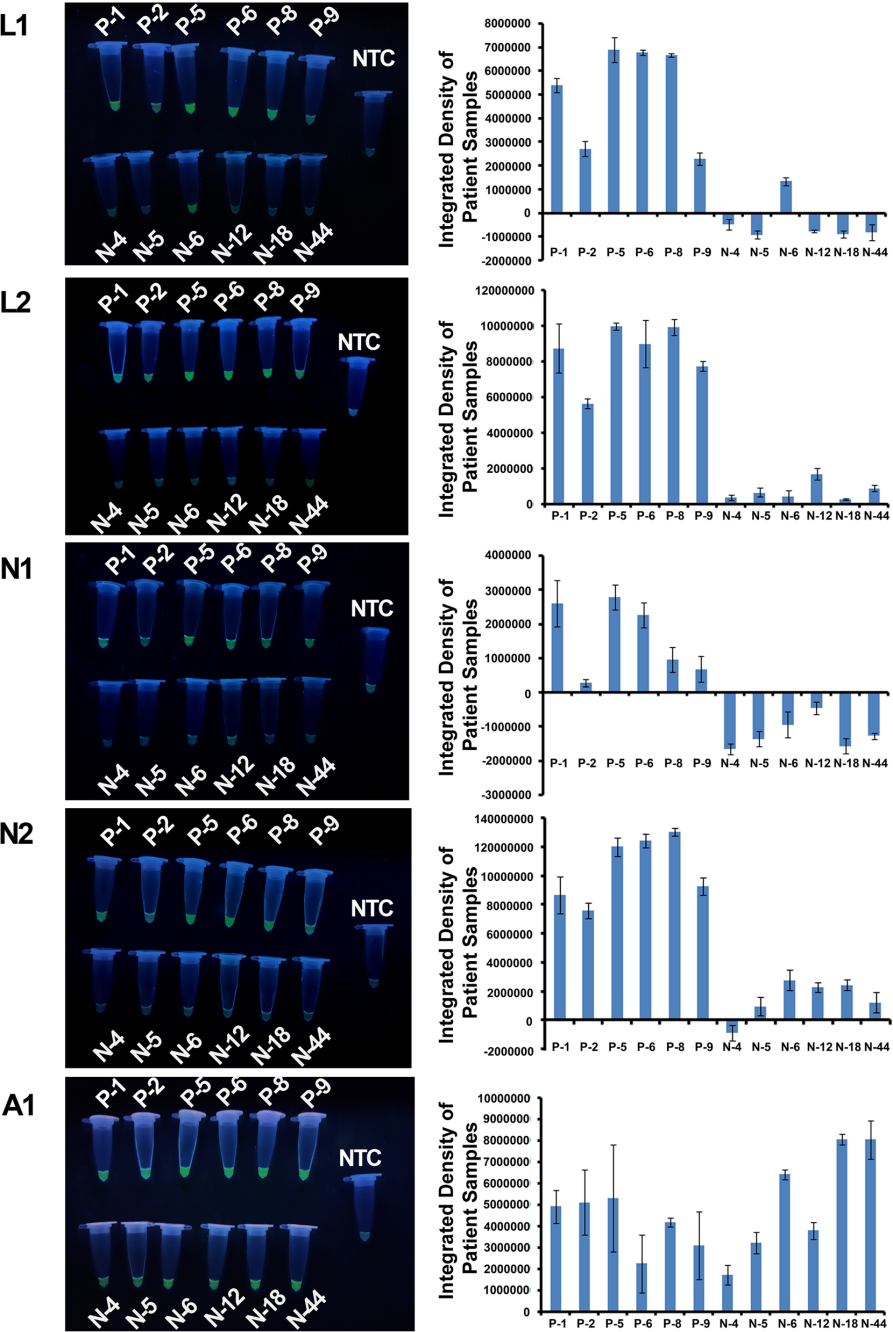
Evaluation of LAMP Primers (L1, L2, N1, N2 and A1 primers sets) with patient samples

Primer sets were tested with synthetic templates (Fig. 3) as well as patient samples using LAMP assay (Fig. 4). With the synthetic templates, the green fluorescence that appeared in reactions with primer sets L1, N2, L2 and N1 resulted in better contrast between the positive reaction and NTC (Fig. 3a), as verified through densitometry of reaction tubes (Fig. 3 c). Further, the amplicons were electrophoresed on a 2% agarose gel and only the positive reactions resulted in a ladder pattern, while the NTCs did not show any detectable amplicons. In order to further test the discriminating efficiency of the primers, the primers that fared well with synthetic templates were evaluated with patient samples. With patient samples, the L1 primer set resulted in one false positive and one of the patient sample (P-2) resulted in only a mild fluorescence (Fig. 4a). N1, N2 and L2 primer sets appeared to have the best contrast between the COVID-19 positive and negative samples as may be observed from the photographs and densitometric ratios (Fig. 4a, b). In view of better discrimination between SARS-CoV2 and other related viruses, L1 was chosen as one of the primer sets for final validation of RT-LAMP assay on a different and more number of patient samples. Additionally, N1 or L2 primer sets were chosen for second assay, because of better contrast between the positive and negative samples and since it did not result in any false negative or false positive. Two loci test employing a combination of L1 and L2 primers or L1 and N1 primers would thus provide a good resolution between the COVID-19 positive and negative samples. A1 and A2 primer sets were used for β-Actin human control gene. A1 primers set appeared to have better contrast than A2 primer set.

### Conservation of primer binding sites in SARS-CoV-2 variants

Analysis was carried out, in order to assess the conservation of primer binding sites and suitability of our LAMP assay for all the WHO notified VOC and VOI of SARS-CoV-2. First 200 sequences of SARS-CoV-2 variants were downloaded from NCBI GenBank. For three variants (B.1.617.1, B.1.617.2 and P.3) lesser than 200 sequences were available in GenBank, hence all were downloaded. Accession numbers of the sequences are available in Additional file 1. The region of genome where L1 and L2 primers were designed was extracted from the complete genomes and multiple sequence alignments were carried out for each region. Only few nucleotides (marked with a star) showed a mismatch in a fraction of sequences, as may be observed in sequence logos generated from the alignments. It was found that the primer binding sites were strongly conserved in all the WHO notified VOC and VOI of SARS-CoV-2 (Additional file 4).

### Validation of RT-LAMP assay

Taqman™ rt-RT-PCR was carried out using the US-CDC protocol primers N1 and N2 on 22 patient samples. RT-LAMP assay was carried out in a double blind manner, using the primer sets L1 and L2 (Table 3). The negatives in either RT-LAMP or rt-RT-PCR are marked with light green color and positives are marked with light red color. For 18 samples, out of 22, the results of RT-LAMP, matched between the L1 and L2 primer sets. For the remaining four samples, where there was a mismatch, a COVID-19 negative call was made on the basis of RT-LAMP assay. Only in one out of these four, the sample gave a positive result with rt-RT-PCR. In total, there were three false negatives in RT-LAMP using the L1 and L2 primer sets, which could be due to low viral load. Optimized RT-LAMP assay protocol was independently verified at a second site at Mumbai by Reliance Industries (RIL), on a much larger set of samples and the result statistics tallied with the data reported here. On other site RIL also carried out Taqman™ rt-RT-PCR on 148 patient samples. RT-LAMP assay was carried out in a double blind manner, using L1 and N1 primer sets (Additional file 5). The negatives in either RT-LAMP or rt-RT-PCR are marked with light green color and positives are marked with light red color. For 132 samples, out of 148, the results of RT-LAMP, matched with Taqman™ rt-RT-PCR results.

**Table 3:**
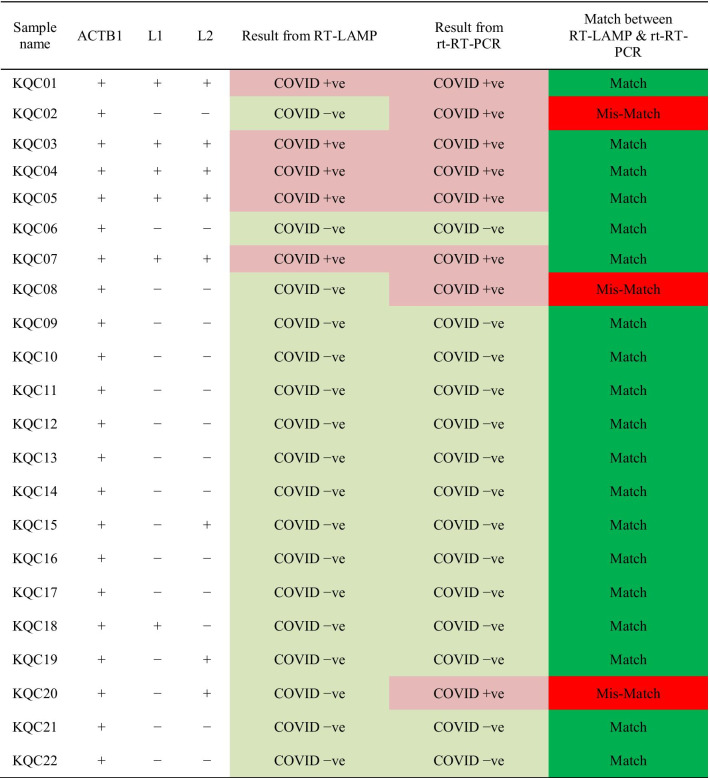
Validation of RT-LAMP assay

## Discussion

As per several reports and speculations, increasing the number of tests may show a significantly higher prevalence of infection [34]. Detection of infected individuals is critical to circumvent the spread of disease and effectuation of containment measures [35, 36]. The problem is accentuated by the fact that a large percentage of infected individuals may not show any symptoms or signs of only a mild infection [8], which may go undetected, especially in rural areas with paucity of medical facilities. Further, in developing and underdeveloped countries with limited resources and State funded testing, this involves a huge cost and burden to the economy [37, 38]. At several places, the expertise and high end instrumentation required for carrying out rt-RT-PCR assays may not be readily available, leading to higher costs in transportation of samples and delays in getting the test results. Hence, cheaper methods for testing individuals, which are also less resource intensive in terms of instrumentation, are always sought after. RT-LAMP can be used as a viable, cheaper and less resource intensive method for testing of COVID-19. RT-LAMP protocols have been developed for the detection of many other viruses, including influenza strains, Zika, Japanese encephalitis, Chikungunya, West Nile virus, etc. [27–30]. Several researchers have also developed RT-LAMP protocols for the detection of COVID-19 [39–42]. Here, we have developed, optimized and validated a two step RT-LAMP method for detection of COVID-19.

A detailed survey of genomic sequences of SARS-CoV2 and other betacoronavirues was carried out and LAMP primers were designed (Fig. 1) in such a manner that they should be able to distinguish SARS-CoV2 from very similar SARS-CoV. For this purpose all the available genomic sequences in the betacoronavirus group were downloaded from NCBI GenBank and primer binding regions were extracted and aligned (Additional file 1). Among the 19 available genomes, the second top BLAST hit for the primer binding sites was in SARS-CoV genome. This was expected considering that both these viruses have jumped from bats to humans and share an overall 80% nucleotide sequence similarity with about 94.6% similarity at protein level, especially in the genes involved in the replication of virus [43, 44]. MERS is another betacoronaviruses that may still be circulating in the population [45, 46]; however the sequence similarity, atleast in our LAMP primer binding sites was very low. It is noteworthy, that atleast one of the outer primers (F3 and B3) of the L1, N1 and RdRp primer set, shows significant differences at the primer binding sites in SARS-CoV2 and closely related SARS-CoV. This is important for accurate testing of COVID-19 and discrimination from SARS-CoV infection. We analyzed the 5642 complete SARS-CoV2 genomes and found that primer binding sites of our LAMP primers are well conserved (Fig. 1, Table 2). Further, L1 primer set lies in NSP1 gene, which was recently shown to be highly expressed in nasopharyngeal and saliva samples from more than 100 patients [47]. L1 and L2 primer binding sites are conserved in all the WHO notified VOC and VOI. Thus, our RT-LAMP assay may be used for detection of SARS-CoV-2, including the presently globally dominant Delta variant.

Several enzymes that are capable of strand displacement activity have been used in LAMP reactions [48–51]. In order to test which of these DNA polymerases works best and is easier and relatively inexpensive, for our assays, we tested the wild type enzymes: Klenow fragment, Bsm large fragment, Bst large fragment as well as engineered variants such as Bst 2.0 and Bst 3.0 (Fig. 2, Additional file 3) that have been shown to have improved speed of reaction and ability to withstand inhibitors that are commonly found in biological specimens (https://international.neb.com/products/m0374-bst-3-0-dna-polymerase). Klenow fragment had a compulsory requirement of denaturation of template at high temperature followed by quick chill, before adding the enzyme, for a successful LAMP reaction (data not shown). All other enzymes did not have this requirement. Further, all other enzymes except Bst 3.0 required addition of Mg +  + ions as MgCl_2_ (Klenow) or MgSO_4_ (Bsm, Bst and Bst 2.0) for efficient LAMP amplification (Additional file 3). The time required for LAMP amplification was highest for Klenow and lowest when Bst 3.0 was used as LAMP polymerase. Using higher temperature for LAMP reaction helps in avoiding non specific amplification, hence it is desirable. Bst 2.0 and Bst 3.0 worked efficiently at higher temperatures as compared to Klenow which worked at 50 °C, hence all further experiments were carried out with Bst 3.0 polymerase.

Detection of amplification in RT-LAMP reaction may be carried out by a realtime PCR, electrophoresing the amplicons on an agorose gel, colorimetric detection of pH changes during the amplification or by addition of DNA binding fluorescent dyes in the LAMP reaction [25, 26, 28]. Several fluorescent dyes such as SYBR Green I, Eva Green, SYTO 9, etc. have been used for this purpose. We have used SYBR Green I in our assays. For Bst 2.0 and Bst 3.0 addition of only 0.2 µL of 1:100 diluted SYBR Green I dye was found to be sufficient for naked eye detection of successful LAMP amplification (Fig. 2).

On the basis of better results and conservation of primer binding sites in all VOC and VOI of SARS-CoV-2, the L1 and L2 primer set were selected for validation on patient samples, in a double blind study. We found that there were three false negatives in RT-LAMP assay when compared to the Taqman™ rt-RT-PCR assay (Table 3). In the study carried out by RIL on validation of RT-LAMP assay, it was observed that out of 148 patient samples, the results of 15 samples were not matching with Taqman™ rt-RT-PCR assay. This could be due to lower viral load in these samples. Recently it was shown that on 8th day after exposure and establishment of SARS-CoV2 infection, there was 38% chance of false negative detection in rt-RT-PCR test, which was minimal. The percentage of false negative detection was nearly 100% on day 1 and reduced to 67% by day 4. The issue of false negative may be overcome by testing the individuals at least two times at an interval of 3–4 days [52].

## Conclusion

We found that our RT-LAMP assay was 89.2% accurate when compared to the current gold standard Taqman™ rt-RT-PCR assay. We further conclude that our assay may be used for detection of all current WHO notified VOC and VOI of SARS-CoV-2. It is also pertinent to mention that RT-LAMP assay can be used as a feasible, low cost, less resource, alternative molecular diagnostic test for COVID-19. It may be used as a primary assay as well as for routine surveillance in schools, offices and other organizations, especially in rural or far-flung areas where elaborate instrumentation facilities do not exist.

## Supplementary Information

**Additional file 1**: All the reference sequences (NCBI GenBank RefSeq) that were available for betacoronaviruses and BLAST results of all LAMP primer binding sites.

**Additional file 2**: Mapping of identified sequence variants in the primer binding sites.

**Additional file 3**: Optimization conditions for Lamp reaction.

**Additional file 4**: Conservation of L1 and L2 primer binding sites in SARS-CoV-2 variants

**Additional file 5**: Validation of RT-LAMP Assay using patient samples (RIL, Mumbai, India).

## Data Availability

All the datasets used and/or analysis during the current study are available as figures, tables and supplementary files. Any addition information may be obtained from the corresponding author on a reasonable request.

## Abbreviations

SARS-CoV2: Severe Acute Respiratory Syndrome Coronavirus 2
COVID-19: Coronavirus disease 2019
VOC: Variants of concern
VOI: Variants of interest
RT-LAMP: Reverse Transcription Loop-mediated isothermal AMPlification
MERS: Middle East Respiratory Syndrome
rt-RT-PCR: Reverse transcription real-time PCR
RdRp: RNA-dependent RNA polymerase
E: Envelope protein
N: Nucleocapsid protein
US-CDC: United States Centres for Disease Control
NGS: Next generation sequencing
WHO: World Health Organization
US-FDA: United States Food and Drug Administration
VTM: Viral transport medium
PANC-1: Pancreatic cancer cell line
DMEM: Dulbecco's modified eagle medium
FBS: Fetal bovine serum
DMSO: Dimethyl sulfoxide
