# Supplementary figures and images for: Optimization and validation of RT-LAMP assay for diagnosis of SARS-CoV2 including the globally dominant Delta variant
Source: Virol J. 2021 Aug 30;18:178. doi: 10.1186/s12985-021-01642-9 (PMC8404189; doi:10.1186/s12985-021-01642-9)

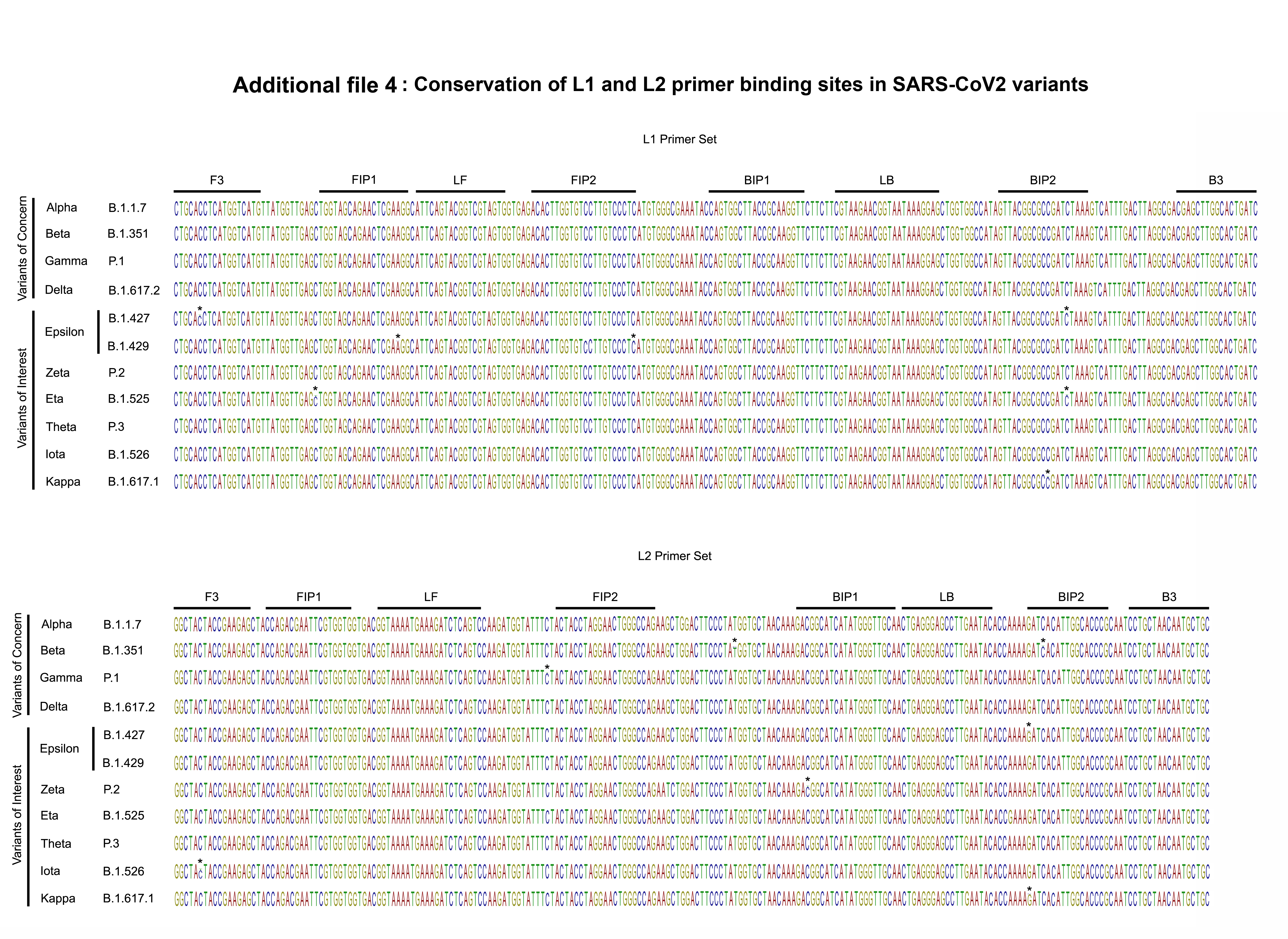

Supplement: Supplementary file 4 — Additional file 4: Conservation of L1 and L2 primer binding sites in SARS-CoV-2 variants [file 12985_2021_1642_MOESM4_ESM.jpg]
