# Supplementary material for: Optimization and validation of RT-LAMP assay for diagnosis of SARS-CoV2 including the globally dominant Delta variant
Source: Virol J. 2021 Aug 30;18:178. doi: 10.1186/s12985-021-01642-9 (PMC8404189; doi:10.1186/s12985-021-01642-9)
Supplement: Supplementary file 5 — Additional file 5: Validation of RT-LAMP Assay using patient samples (RIL, Mumbai, India). [file 12985_2021_1642_MOESM5_ESM.docx]

**Additional file 5: Validation of RT-LAMP Assay (RIL, Mumbai, India)**

| Sample Name | **L1** | **N1** | **Result from RT-LAMP** | **Result from**  **rt-RT-PCR** | **Match Between RT-LAMP & rt-RT-PCR** |
| --- | --- | --- | --- | --- | --- |
|  |  |  |  |  |  |
| RIL1 | + | + | COVID +ve | COVID +ve | Match |
| RIL2 | + | + | COVID +ve | COVID +ve | Match |
| RIL3 | + | + | COVID +ve | COVID +ve | Match |
| RIL4 | + | + | COVID +ve | COVID +ve | Match |
| RIL5 | + | + | COVID +ve | COVID +ve | Match |
| RIL6 | + | + | COVID +ve | COVID +ve | Match |
| RIL7 | + | + | COVID +ve | COVID +ve | Match |
| RIL8 | + | + | COVID +ve | COVID +ve | Match |
| RIL9 | + | + | COVID +ve | COVID +ve | Match |
| RIL10 | + | + | COVID +ve | COVID +ve | Match |
| RIL11 | + | + | COVID +ve | COVID +ve | Match |
| RIL12 | + | + | COVID +ve | COVID +ve | Match |
| RIL13 | + | + | COVID +ve | COVID +ve | Match |
| RIL14 | + | + | COVID +ve | COVID +ve | Match |
| RIL15 | + | + | COVID +ve | COVID +ve | Match |
| RIL16 | + | + | COVID +ve | COVID +ve | Match |
| RIL17 | + | + | COVID +ve | COVID +ve | Match |
| RIL18 | + | + | COVID +ve | COVID +ve | Match |
| RIL19 | + | + | COVID +ve | COVID +ve | Match |
| RIL20 | + | + | COVID +ve | COVID +ve | Match |
| RIL21 | + | + | COVID +ve | COVID +ve | Match |
| RIL22 | + | + | COVID +ve | COVID +ve | Match |
| RIL23 | + | + | COVID +ve | COVID +ve | Match |
| RIL24 | + | + | COVID +ve | COVID +ve | Match |
| RIL25 | + | + | COVID +ve | COVID +ve | Match |
| RIL26 | - | - | COVID -ve | COVID -ve | Match |
| RIL27 | - | - | COVID -ve | COVID -ve | Match |
| RIL28 | - | - | COVID -ve | COVID -ve | Match |
| RIL29 | - | - | COVID -ve | COVID -ve | Match |
| RIL30 | - | - | COVID -ve | COVID -ve | Match |
| RIL31 | - | - | COVID -ve | COVID -ve | Match |
| RIL32 | - | - | COVID -ve | COVID -ve | Match |
| RIL33 | - | - | COVID -ve | COVID -ve | Match |
| RIL34 | - | - | COVID -ve | COVID -ve | Match |
| RIL35 | - | - | COVID -ve | COVID -ve | Match |
| RIL36 | - | - | COVID -ve | COVID -ve | Match |
| RIL37 | - | - | COVID -ve | COVID -ve | Match |
| RIL38 | - | - | COVID -ve | COVID -ve | Match |
| RIL39 | + | + | COVID +ve | COVID +ve | Match |
| RIL40 | - | - | COVID -ve | COVID +ve | Mis-Match |
| RIL41 | + | + | COVID +ve | COVID +ve | Match |
| RIL42 | + | + | COVID +ve | COVID +ve | Match |
| RIL43 | + | + | COVID +ve | COVID +ve | Match |
| RIL44 | + | + | COVID +ve | COVID +ve | Match |
| RIL45 | + | + | COVID +ve | COVID +ve | Match |
| RIL46 | + | + | COVID +ve | COVID +ve | Match |
| RIL47 | + | - | Inconclusive | COVID -ve | Mis-Match |
| RIL48 | - | - | COVID -ve | COVID -ve | Match |
| RIL49 | - | - | COVID -ve | COVID -ve | Match |
| RIL50 | - | - | COVID -ve | COVID -ve | Match |
| RIL51 | - | - | COVID -ve | COVID -ve | Match |
| RIL52 | - | - | COVID -ve | COVID -ve | Match |
| RIL53 | + | - | Inconclusive | COVID -ve | Mis-Match |
| RIL54 | - | - | COVID -ve | COVID -ve | Match |
| RIL55 | - | + | Inconclusive | COVID -ve | Mis-Match |
| RIL56 | + | + | COVID +ve | COVID -ve | Mis-Match |
| RIL57 | + | + | COVID +ve | COVID -ve | Mis-Match |
| RIL58 | NYD | - | COVID -ve | COVID -ve | Match |
| RIL59 | NYD | - | COVID -ve | COVID -ve | Match |
| RIL60 | NYD | - | COVID -ve | COVID -ve | Match |
| RIL61 | NYD | - | COVID -ve | COVID -ve | Match |
| RIL62 | NYD | - | COVID -ve | COVID -ve | Match |
| RIL63 | NYD | + | COVID +ve | COVID +ve | Match |
| RIL64 | NYD | - | COVID -ve | COVID -ve | Match |
| RIL65 | NYD | - | COVID -ve | COVID -ve | Match |
| RIL66 | NYD | - | COVID -ve | COVID -ve | Match |
| RIL67 | NYD | - | COVID -ve | COVID -ve | Match |
| RIL68 | NYD | - | COVID -ve | COVID -ve | Match |
| RIL69 | NYD | - | COVID -ve | COVID -ve | Match |
| RIL70 | NYD | - | COVID -ve | COVID -ve | Match |
| RIL71 | NYD | + | COVID +ve | COVID +ve | Match |
| RIL72 | NYD | + | COVID +ve | COVID -ve | Mis-Match |
| RIL73 | NYD | - | COVID -ve | COVID -ve | Match |
| RIL74 | NYD | - | COVID -ve | COVID -ve | Match |
| RIL75 | NYD | - | COVID -ve | COVID -ve | Match |
| RIL76 | NYD | - | COVID -ve | COVID -ve | Match |
| RIL77 | NYD | - | COVID -ve | COVID -ve | Match |
| RIL78 | NYD | - | COVID -ve | COVID -ve | Match |
| RIL79 | NYD | - | COVID -ve | COVID +ve | Mis-Match |
| RIL80 | NYD | - | COVID -ve | COVID -ve | Match |
| RIL81 | NYD | - | COVID -ve | COVID -ve | Match |
| RIL82 | NYD | - | COVID -ve | COVID -ve | Match |
| RIL83 | NYD | - | COVID -ve | COVID -ve | Match |
| RIL84 | NYD | - | COVID -ve | COVID -ve | Match |
| RIL85 | NYD | - | COVID -ve | COVID -ve | Match |
| RIL86 | NYD | - | COVID -ve | COVID -ve | Match |
| RIL87 | NYD | + | COVID +ve | COVID +ve | Match |
| RIL88 | NYD | - | COVID -ve | COVID -ve | Match |
| RIL89 | NYD | - | COVID -ve | COVID -ve | Match |
| RIL90 | NYD | + | COVID -ve | COVID +ve | Mis-Match |
| RIL91 | NYD | + | COVID +ve | COVID +ve | Match |
| RIL92 | NYD | + | COVID +ve | COVID +ve | Match |
| RIL93 | NYD | + | COVID +ve | COVID +ve | Match |
| RIL94 | NYD | + | COVID +ve | COVID +ve | Match |
| RIL95 | NYD | + | COVID +ve | COVID +ve | Match |
| RIL96 | NYD | + | COVID +ve | COVID +ve | Match |
| RIL97 | NYD | + | COVID +ve | COVID +ve | Match |
| RIL98 | NYD | - | COVID -ve | COVID -ve | Match |
| RIL99 | NYD | - | COVID -ve | COVID -ve | Match |
| RIL100 | NYD | - | COVID -ve | COVID -ve | Match |
| RIL101 | NYD | - | COVID -ve | COVID -ve | Match |
| RIL102 | NYD | - | COVID -ve | COVID -ve | Match |
| RIL103 | NYD | - | COVID -ve | COVID -ve | Match |
| RIL104 | NYD | - | COVID -ve | COVID -ve | Match |
| RIL105 | NYD | - | COVID -ve | COVID -ve | Match |
| RIL106 | NYD | - | COVID -ve | COVID -ve | Match |
| RIL107 | NYD | - | COVID -ve | COVID -ve | Match |
| RIL108 | NYD | + | COVID +ve | COVID +ve | Match |
| RIL109 | NYD | + | COVID +ve | COVID +ve | Match |
| RIL110 | NYD | + | COVID +ve | COVID +ve | Match |
| RIL111 | NYD | + | COVID +ve | COVID +ve | Match |
| RIL112 | NYD | + | COVID +ve | COVID +ve | Match |
| RIL113 | NYD | + | COVID +ve | COVID +ve | Match |
| RIL114 | NYD | + | COVID +ve | COVID +ve | Match |
| RIL115 | NYD | + | COVID +ve | COVID +ve | Match |
| RIL116 | NYD | + | COVID +ve | COVID +ve | Match |
| RIL117 | NYD | + | COVID +ve | COVID +ve | Match |
| RIL118 | NYD | + | COVID +ve | COVID +ve | Match |
| RIL119 | NYD | + | COVID +ve | COVID +ve | Match |
| RIL120 | NYD | - | COVID -ve | COVID -ve | Match |
| RIL121 | NYD | - | COVID -ve | COVID -ve | Match |
| RIL122 | NYD | - | COVID -ve | COVID -ve | Match |
| RIL123 | NYD | - | COVID -ve | COVID -ve | Match |
| RIL124 | NYD | - | COVID -ve | COVID -ve | Match |
| RIL125 | NYD | - | COVID -ve | COVID -ve | Match |
| RIL126 | NYD | - | COVID -ve | COVID -ve | Match |
| RIL127 | NYD | - | COVID -ve | COVID -ve | Match |
| RIL128 | NYD | + | COVID +ve | COVID -ve | Mis-Match |
| RIL129 | NYD | + | COVID +ve | COVID +ve | Match |
| RIL130 | NYD | + | COVID +ve | COVID +ve | Match |
| RIL131 | NYD | + | COVID +ve | COVID +ve | Match |
| RIL132 | NYD | + | COVID +ve | COVID +ve | Match |
| RIL133 | NYD | + | COVID +ve | COVID +ve | Match |
| RIL134 | NYD | + | COVID +ve | COVID +ve | Match |
| RIL135 | NYD | + | COVID +ve | COVID +ve | Match |
| RIL136 | NYD | - | COVID -ve | COVID +ve | Mis-Match |
| RIL137 | NYD | + | COVID +ve | COVID +ve | Match |
| RIL138 | NYD | + | Low | COVID +ve | Match |
| RIL139 | NYD | - | NA | COVID +ve | **Sample Lost** |
| RIL140 | NYD | + | COVID +ve | COVID +ve | Match |
| RIL141 | NYD | - | COVID -ve | COVID -ve | Match |
| RIL142 | NYD | + | COVID +ve | COVID -ve | Mis-Match |
| RIL143 | NYD | + | COVID +ve | COVID -ve | Mis-Match |
| RIL144 | NYD | + | COVID +ve | COVID -ve | Mis-Match |
| RIL145 | NYD | - | COVID -ve | COVID -ve | Match |
| RIL146 | NYD | - | COVID -ve | COVID -ve | Match |
| RIL147 | NYD | + | COVID +ve | COVID -ve | Mis-Match |
| RIL148 | NYD | - | COVID -ve | COVID -ve | Match |
